# Keratin 5 basal cells are temporally regulated developmental and tissue repair progenitors in bladder urothelium

**DOI:** 10.1152/ajprenal.00378.2023

**Published:** 2024-04-18

**Authors:** Brian Becknell, Mohammad El-Harakeh, Felipe Rodriguez-Tirado, Kelly M. Grounds, Birong Li, Macie Kercsmar, Xin Wang, Ashley R. Jackson

**Affiliations:** ^1^Kidney and Urinary Tract Center, Nationwide Children's Hospital, Columbus, Ohio, United States; ^2^Division of Nephrology and Hypertension, Nationwide Children’s Hospital, Columbus, Ohio, United States; ^3^Department of Pediatrics, The Ohio State University College of Medicine, Columbus, Ohio, United States

**Keywords:** bladder urothelium, cyclophosphamide, keratin 5 urothelial cell, organoid, progenitor

## Abstract

Urothelium forms a distensible yet impermeable barrier, senses and transduces stimuli, and defends the urinary tract from mechanical, chemical, and bacterial injuries. Biochemical and genetic labeling studies support the existence of one or more progenitor populations with the capacity to rapidly regenerate the urothelium following injury, but slow turnover, a low mitotic index, and inconsistent methodologies obscure progenitor identity. The progenitor properties of basal keratin 5 urothelial cells (K5-UCs) have been previously investigated, but those studies focused on embryonic or adult bladder urothelium. Urothelium undergoes desquamation and apoptosis after birth, which requires postnatal proliferation and restoration. Therefore, we mapped the fate of bladder K5-UCs across postnatal development/maturation and following administration of cyclophosphamide to measure homeostatic and reparative progenitor capacities, respectively. In vivo studies demonstrate that basal K5-UCs are age-restricted progenitors in neonates and juveniles, but not in adult mice. Neonatal K5-UCs retain a superior progenitor capacity in vitro, forming larger and more differentiated urothelial organoids than adult K5-UCs. Accordingly, K5-UC transcriptomes are temporally distinct, with enrichment of transcripts associated with cell proliferation and differentiation in neonates. Induction of urothelial proliferation is sufficient to restore adult K5-UC progenitor capacity. Our findings advance the understanding of urothelial progenitors and support a linear model of urothelial formation and regeneration, which may have significant impact on therapeutic development or tissue engineering strategies.

**NEW & NOTEWORTHY** Fate mapping reveals an important linear relationship, whereby bladder basal urothelial cells give rise to intermediate and superficial cells in an age-restricted manner and contribute to tissue repair. Neonatal basal cells reprise their role as superior progenitors in vitro and display distinct transcriptional signatures, which suggest progenitor function is at least partially cell intrinsic. However, the urothelium progenitor niche cannot be overlooked, since FGF7 rescues adult basal cell progenitor function.

## Graphical abstract

**Figure:**
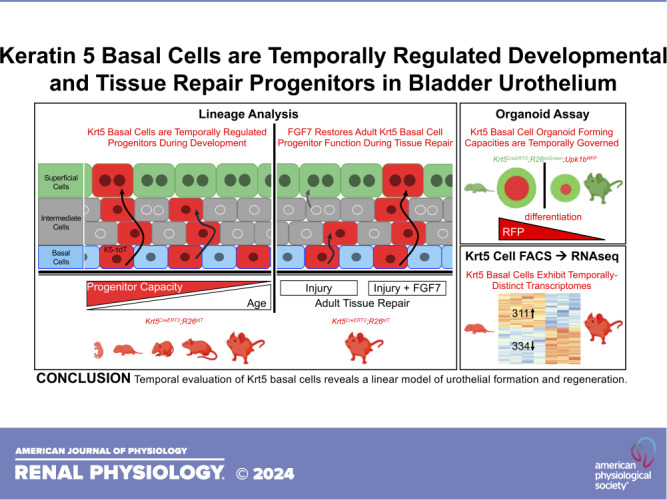

## INTRODUCTION

Urothelium forms an impermeable and distensible urine-blood barrier, with complex sensory and signaling functions (1). Organized into basal, intermediate, and superficial cell layers, urothelial cells exhibit progressive levels of differentiation (1, 2). Terminally differentiated superficial cells are large multinucleated postmitotic cells that confer most of the functions required of urothelium. Superficial cells elaborate urothelial plaques at their apical membrane and tight junctions with neighboring cells. Together, urothelial plaques and tight junctions make bladder urothelium the most impermeable epithelial lining in the human body (3). Intermediate cells lie just beneath the superficial cell layer and can be subcategorized into binucleated and mononucleated subsets (4). Basal cells are small, line the basement membrane, and represent the largest percentage of urothelial cells in the bladder (5).

The adult bladder urothelium is nearly quiescent, with a proliferative index of 0.01% during homeostasis (2, 6, 7). However, urothelium mounts an impressive proliferation rate and is quickly restored following injury (5, 8–13). The identity of the progenitor responsible for urothelial restoration has been the focus of many investigations, but evidence supporting progenitor cell identification is conflicting (14).

Modern lineage analysis strategies use Cre;LoxP technology, where a Cre recombinase-driver strain is crossed with a stop-floxed reporter strain [reviewed in Jackson et al. (14)]. Cre recombinase can be constitutively or inducibly activated in a cell-specific manner, such that specific urothelial cell subsets can be permanently marked and then evaluated, a method also referred to as genetic fate mapping. The fate of urothelial cell subsets has been evaluated through development and following chemical, bacterial, and surgical injuries. Those studies point to progenitor cells within the basal or intermediate cell layers, which appear to be differentially activated during homeostasis and injury (5, 12, 13, 15–17). These disparate progenitor theories assert either a linear model, with basal cells forming intermediate cells which then form superficial cells (B→I→S), or a nonlinear model, with basal cells forming only basal cells and intermediate cells forming intermediate and superficial cells (B→B; I→S).

Keratin 5 (Krt5/K5) is considered a basal urothelial cell maker in the bladder, although some intermediate cells also express K5 (5). K5 basal cells are thought to contain a urothelial progenitor, yet reports are conflicting (4, 5, 13). We previously reported that K5 expressing urothelial cells (K5-UCs) in the kidney exhibit a temporally regulated progenitor capacity, whereby neonatal and juvenile but not adult K5-UCs form uroplakin-expressing urothelial cells (Upk-UCs) during postnatal maturation and in response to urinary tract obstruction (18). Despite being recognized as a slow turnover epithelium in adults, the neonatal bladder urothelium is markedly proliferative until *postnatal* *day 10*, likely as a consequence of an overlapping wave of apoptosis and desquamation through the first 9 days (7, 19, 20). Most progenitor studies focus on embryonic development, or adult urothelium, largely overlooking the dynamic features of postnatal bladder urothelium.

Understanding that the bladder urothelium is highly dynamic during postnatal life, we set out to define the fate of K5-UCs labeled at various postnatal stages to define how the Krt5 lineage contributes to urothelial maturation and how those populations contribute to tissue repair. Rather than the existence of distinct basal and intermediate cell progenitor populations, our data indicate that these populations are developmentally linked and support a linear model of urothelial formation and regeneration (B→I→S).

## MATERIALS AND METHODS

### Animals

Public Health Service Animal Welfare Assurance No. A3544-01 and Institutional Animal Care and Use Committee No. AR16-00058 were used. The *Krt5*^CreERT2^ (No. 018394) tamoxifen-inducible Cre driver was purchased from Jackson Laboratories (21). The *Rosa26*^tdT^ (called *R26*^tdT^, No. 007909) and *Rosa26*^zsGreen^ (called *R26*^zsGreen^, No. 007906) Cre reporter lines were purchased from Jackson Laboratories (22). *Upk1b*^RFP^ mice were maintained in our breeding colony (23, 24). Mouse lines and genotyping information are detailed in Supplemental Table S1. Cre recombination was induced using a single intraperitoneal dose of tamoxifen (TMX; 75 mg/kg body wt in corn oil, Sigma, St. Louis, MO) at precise postnatal (P) time points (18). Mice were euthanized at P42 for evaluation of the homeostatic urothelial progenitor cell or subjected to cyclophosphamide (CYC) treatment (methods described below) for the evaluation of the injury-induced, tissue repair UC progenitor.

### Lineage Analysis

For each genetic fate mapping procedure, baseline samples were evaluated by harvesting bladders 24 h after TMX administration (18). Reporter specificity (i.e., Krt5^+^;tdT^+^/total tdT^+^ cells) and labeling efficiency (i.e., Krt5^+^;tdT^+^/total Krt5^+^ cells) were confirmed by immunofluorescent labeling (18). The fate of inducibly labeled K5-UCs was evaluated in tissues harvested at P42 (adult). Unexpected TMX effects were ruled out by using Cre-negative;*R26*^tdT^ mice, and inappropriate Cre;LoxP recombination was ruled by comparing corn oil-treated Cre-positive;*Rosa26* mice.

### Microscopy

Formalin-fixed paraffin-embedded tissues were sectioned at 3–5 µm and used for routine staining and immunolocalization. Hematoxylin and eosin staining was performed as previously described (18, 25).

### Immunolocalization

Following deparaffinization and rehydration, antigen retrieval was achieved by submerging slides in sodium citrate buffer in a pressure cooker. Nonspecific binding was blocked using SuperBlock (ScyTek Laboratories, Logan, UT). Immunolocalization was performed using anti-Fabp4, anti-Ki67, anti-Krt5, anti-Krt14, anti-pErk1/2, anti-pFrs2a, anti-RFP, anti-tdT, anti-Upk3a, and anti-zsGreen primary antibodies (Supplemental Table S2) and Cy3-, Alexa Fluor 488-, and AMCA- and Cy5-conjugated species-specific secondary antibodies (1:300, Jackson ImmunoResearch Laboratories, West Grove, PA). Coverslips were mounted using Vectashield ± DAPI (Vector Laboratories, Burlingame, CA), and images were captured using an Olympus BX51 microscope equipped with a CX9000 camera (Olympus Corporation, Tokyo, Japan). All micrographs underwent equivalent brightness and contrast adjustments to enhance print view. Random high-powered fields were selected from a 125 × 125 µm grid for evaluation and quantitation. Quantitation of UC expression was performed by establishing a total cell count (using DAPI, which marked nuclei) and cell-specific expression markers (Krt5, Upk, Fabp4, tdT, EdU, and Krt14), using cell counting tools available in StereoInvestigator software or QuPath software (v. 0.4.3) (18, 26). Graphs were generated, and statistical analyses were performed using GraphPad Prism software (v. 9.5.1).

### EdU Incorporation Assay

5-Ethynyl-20-deoxyuridine (EdU) incorporation assays were used to measure urothelial cell proliferation (18, 24). Briefly, 250 µg was administered via intraperitoneal injection at the indicated time points. Mice were euthanized after 2 h, bladders were formalin-fixed and paraffin-embedded, and tissues were sectioned at 5 µm. EdU detection was performed using the Click-iT EdU Alexa Fluor 488 Imaging kit (Life Technologies, Carlsbad, CA). Quantitative analysis was performed using StereoInvestigator software as aforementioned.

### Cyclophosphamide-Induced Urothelial Injury

To induce urothelial injury, a single ip dose of CYC (150 mg/kg body wt, Sigma-Aldrich, St. Louis, MO) or saline was administered at P42. CYC-induced bladder urothelium injury and restoration was confirmed at 2, 7, and 14 days. Mice were euthanized after 14 days, and bladders were formalin-fixed and paraffin-embedded for analysis.

### Limiting Cell RNA-Sequencing

To evaluate the transcriptome of neonatal and adult K5-UCs, we deployed a limiting cell RNA-sequencing (lcRNAseq) workflow (27). Female bladders were harvested in ice cold 1X PBS, everted with blunt forceps, and incubated with Dispase II (2.5 mg/mL, Sigma-Aldrich, St. Louis, MO) in 1X PBS/50 mM HEPES for 1 h at 37°C. Urothelium was scraped, disaggregated in TrypLE Express (Sigma-Aldrich, St. Louis, MO), then passed through a 70 µm strainer. Red blood cells were lysed using ammonium-chloride-potassium (ACK; ThermoFisher, Waltham, MA) lysis solution, and cells were resuspended in 1X PBS containing 2% fetal bovine serum (FBS, ThermoFisher, Waltham, MA). The BD Influx System (BD Biosciences, San Jose, CA) was used to sort 300 tdT^K5^-UCs from neonatal (TMX^P5^→FACS^P7^) and adult (TMX^P35^→FACS^P42^) *Krt5*^CreERT2^;*R26*^tdT^ mouse bladders. Cells were sorted directly into the Takara SMART-Seq HT lysis buffer containing RNase Inhibitor + 3′ SMART-Seq CDS Primer II A (SMART-Seq HT kit, Takara Bio USA, Ann Arbor, MI). Samples were immediately frozen on dry ice, stored at −80°C, then transported to The Ohio State University Comprehensive Cancer Center NextGen Sequencing Shared Resource facility. lcRNAseq libraries were generated using the Takara SMART-Seq HT kit, Nextera XT DNA Library Preparation and Nextera XT Index kits (Illumina, San Diego, CA). Samples were evaluated using a High Sensitivity DNA Bioanalyzer and Tapestation HS-DNA1000 Bioanalyzer (Agilent), and Qubit (ThermoFisher). Sequencing was performed using a NovaSeq6000 SP 200 cycle (≤2 × 100 bp) flow cell. Data have been deposited in Gene Expression Omnibus (GEO Accession No. GSE248463).

### lcRNAseq Analysis

Demultiplexed FASTQ files were utilized for a comprehensive analysis pipeline encompassing data normalization, differential gene expression detection, and functional enrichment. We obtained the mm10 mouse genome and its corresponding reference gene set from UCSC (https://hgdownload.soe.ucsc.edu/goldenPath/mm10/bigZips/). Adapter sequences were removed, and high-quality filtered reads were generated using Trim Galore v0.6.6. (28). Tophat v2.1.2 (29) was used to map these high-quality reads to the mm10 genome. Successfully mapping reads were sorted, and unique mapped reads were retained by SAMtools v1.15 (30). Quantification of reads per gene was performed by Htseq-count v0.12.4 (31) with a union gene region parameter. EdgeR_3.42.4 (29) was used to identify differentially expressed genes (DEGs). We filtered out any gene that is sparsely quantified in samples with loose criteria, i.e., a total count less than five across all samples, and more than two sample with cpm greater than 0.5. Then, quantile normalization method was used to eliminate or minimize technical variability. Principal component analysis (PCA) was used as an unsupervised dimensionality for gene abundance levels across samples. We defined DEGs as those with a fold change greater than 1.5 and an adjusted *P* value smaller than 0.05. Gene ontology (GO) analysis for the functional enrichments of upregulated and downregulated genes was performed by clusterProfiler_4.8.2 (32). A Chi-square test was used to test for statistically enriched pathways and an FDR of <0.05 was used as the significance threshold to detect enriched molecular functions, biological processes, and cellular components. Finally, transcription factors and their activities were predicted and estimated by decoupleR 2.5.3 (33). For data visualization, we mainly used a series of R packages such as “ggplot2,” “enrichplot,” “pheamap” to create heatmaps, volcano plots, bar plots, and Venn diagrams.

### Western Blot

Bladders were homogenized in 1X RIPA buffer (RPI, Mount Prospect, IL) containing Halt Protease and Phosphatase Inhibitor Single-Use Cocktail, EDTA-Free (Fisher Scientific) using a Qiagen TissueLyser II. Total proteins were then quantified using a DC protein assay (Bio-Rad Laboratories, Hercules, CA). Proteins (20 µg) were resolved by sodium dodecylsulfate–polyacrylamide gel electrophoresis (SDS-PAGE), followed by semi-dry transfer using the Bio-Rad Trans-Blot Turbo Transfer system, with Trans-blot turbo 5X transfer buffer with 20% ethanol and mini-size LF PVDF membranes (Bio-Rad). Blots were blocked using EveryBlot blocking buffer (Bio-Rad), incubated with primary antibodies, including anti-Erk1/2 (Erk), anti-pErk1/2 (pErk), anti-pFrs2 and anti-Gapdh (Supplemental Table S2), StarBright Blue Goat Anti-Species IgG antibodies (1:2500, Bio-Rad), and washed with 0.05% Tween 20 in PBS. Blots were imaged using the ChemiDoc Imaging System (Bio-Rad). Proteins were quantified by densitometry using ImageJ, graphed, and analyzed using GraphPad Prism software (v. 9.5.1).

### Organoid-Forming Assays

Organoid assays were used to examine progenitor capacity in vitro. In brief, bladders were harvested in ice cold 1X PBS, everted with blunt forceps, and incubated with Dispase II (2.5 mg/mL, Sigma-Aldrich, St. Louis, MO) in 1X PBS/HEPES for 1 h at 37°C. Urothelium was stripped from the tissue mechanically, further disaggregated using TrypLE Express (Sigma-Aldrich, St. Louis, MO). Red blood cells were lysed using ACK lysis solution (ThermoFisher, Waltham, MA), and cells were passed through a 70 µm strainer. Cells were resuspended in DMEM-F/12 (ThermoFisher) media containing a cocktail of additives which included; recombinant human fibroblast growth factor 7 (rhFGF7 0.01%, Peprotech, Cranbury, NJ), rhFGF10 (0.02%, Peprotech, Cranbury, NJ), A83-01 (0.02%, Stemcell technologies, BC, Canada), B-27 Supplement (2%, ThermoFisher, Waltham, MA), and amphotericin B (1%, ThermoFisher, Waltham, MA) and mixed with Matrigel [No. 354234 (7.65 mg/mL) Corning, Corning, NY]. Single-cell suspensions were plated in 10 µL Matrigel domes (1,000 cells/Matrigel dome). Media was exchanged every other day, and the approximately 1 mm thick Matrigel dome was imaged using a Nikon Ti2-E microscope (Nikon Instruments Inc., Melville, NY), equipped with a Hamamatsu ORCA-Fusion camera (Hamamatsu Japan). Images were flattened and exported to ImageJ for analysis. Figure legends indicate the number of technical and experimental replicates, the sex and whether sexes were pooled for each experiment. The total number and size of organoids formed per 10 µL Matrigel dome were enumerated. Organoids generated using *Krt5*^CreERT2^;*R26*^zsG^;*Upk1b*^RFP^ mice were analyzed using QuPath software (v. 0.4.3) (26). Briefly, a classifier was applied to detect the boundaries of zsGreen+ (350 threshold constant) organoids and to measure the organoid area and the area within each organoid where RFP (300 threshold constant) was detected. These parameters were used to count organoids, measure organoid size, and measure organoid differentiation (using RFP expression). Five biological replicates were used for each group (neonate and adult). Graphs were generated and statistical analyses were performed using GraphPad Prism software (v. 9.5.1). In some cases, organoids were fixed prior to imaging. Briefly, media was aspirated, and ice cold 4% PFA was added for 30 min, before being exchanged with 1X PBS. In other cases, PFA-fixed organoids were collected in Histogel (Fisher Scientific), and paraffin embedded for slide mounting and immunofluorescent localization.

### FGF7-Induced Urothelial Cell Proliferation

To induce K5-UC proliferation, a single ip dose of rhFGF7 (Palifermin/Kepivance, 5 mg/kg body wt, SOBI, Solna, Sweden) or water was administered to adult *Krt5*^CreERT2^;*R26*^tdT^ mice. Specificity and efficiency of FGF7-induced proliferation were determined using the EdU incorporation assay (outlined in *EdU Incorporation Assay*). To determine whether FGF7 impacted the capacity for K5-UCs to form intermediate or superficial cells, the tdT reporter was induced using a single intraperitoneal dose of TMX (75 mg/kg body wt) at P35. Twenty-four hours later, FGF7 or water (carrier) was administered. At P42, mice were treated with CYC or PBS (carrier). Mice were euthanized 2 wk after CYC or PBS treatment. Bladders were formalin-fixed and paraffin embedded for analysis.

### Statistical Analyses

Statistical analyses were performed using Prism Software (GraphPad Software, La Jolla, CA). When appropriate, we applied a one-way ANOVA or an unpaired two-tailed *t* test. Differences between groups with *P* < 0.05 were statistically significant. Data are presented as means ± SD. Figures indicate sample size, statistical tests, and relevant multiple comparison correction used.

## RESULTS

### Intermediate and Superficial Cells Are Derived From the Basal K5-UC Lineage in a Temporally Restricted Manner

To examine whether K5-UCs are urothelial progenitors in the postnatal bladder, we used the *Krt5*^CreERT2^;*R26*^tdT^ line to map the fate of K5-UCs that were inducibly and permanently labeled at various time points. TMX was administered at postnatal day (P)1, P7, P14, P21, or P35 to induce Cre;LoxP recombination at the *Rosa26* locus and thereby drive the indelible expression of a fluorescent protein [tandem Tomato (tdT)] in UCs with an active *Krt5* promoter and their subsequent progeny. We first determined the specificity and efficiency of our lineage labeling strategy and then mapped the adult fate of the K5-UC lineage.

**Figure 1.**
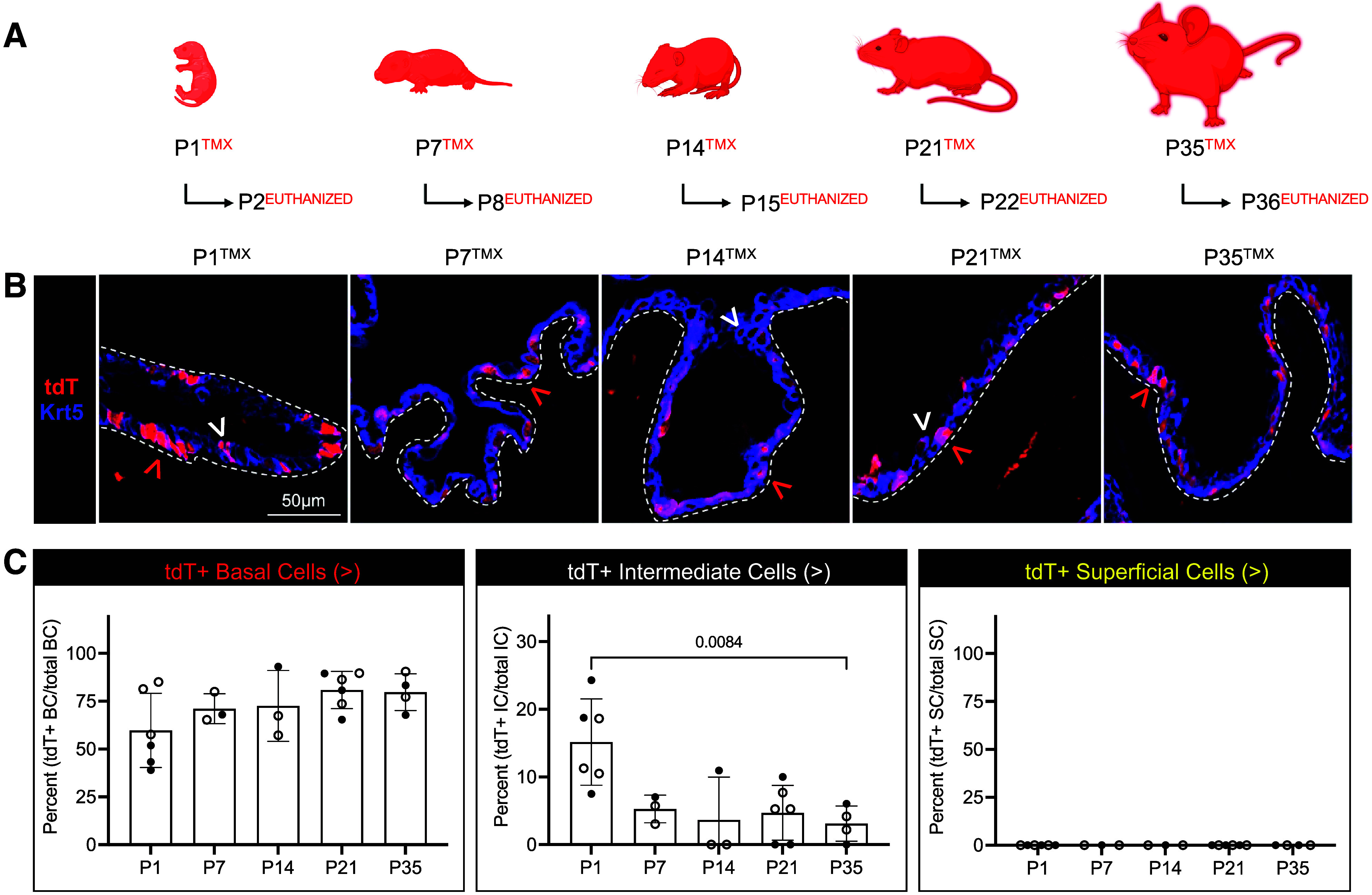
The tdT reporter is efficient and highly specific to K5+ cells. *A*: schematic representation of experimental plan showing the age tamoxifen (TMX) was administered and the age that mice were euthanized. *B*: representative micrographs showing tdT and Krt5 expression in urothelium in each experimental condition. White dashed line: urothelium basement membrane; red arrowhead: tdT+ basal cell; white arrowhead: tdT+ intermediate cell. *C*: graphs showing the percent tdT+ basal, intermediate, and superficial cells at each stage. Bars: mean; error bars: SD; *P* values: one-way ANOVA with Šídák’s multiple comparisons test. P1→P2 (*n* = 6 mice), P7→P8 (*n* = 3 mice), P14→P15 (*n* = 3 mice), P21→P22 (*n* = 5 mice), P35→P36 (*n* = 4 mice). Closed circles: male; open circles: female. Krt5, keratin 5.

Baseline assessments (24 h after TMX administration) demonstrated that tdT labeling was consistently efficient and highly specific across TMX induction stages (Fig. 1 and Supplemental Fig. S1, *A–C*). Our analysis also found that tdT was predominantly expressed by K5-UCs in the basal layer. A subset of K5-UCs that express Krt14 (K14-UCs) are known bladder urothelium progenitors (13). We found that tdT was expressed in a variable fraction of K14-UCs and that K14 indices decreased with age (Supplemental Fig. S1, *D* and *E*). Only rare intermediate cells expressed tdT, and at each instance, tdT localized to a K5+ intermediate cell. No superficial cells expressed tdT in baseline studies (Fig. 1 and Supplemental Fig. S1). These data confirm that the *Krt5*^CreERT2^;*R26*^tdT^ line could be used to efficiently and specifically label K5 basal cells in a temporally controlled manner, and form the foundation for our efforts to map the fate of the basal K5-UCs in this study.

**Figure 2.**
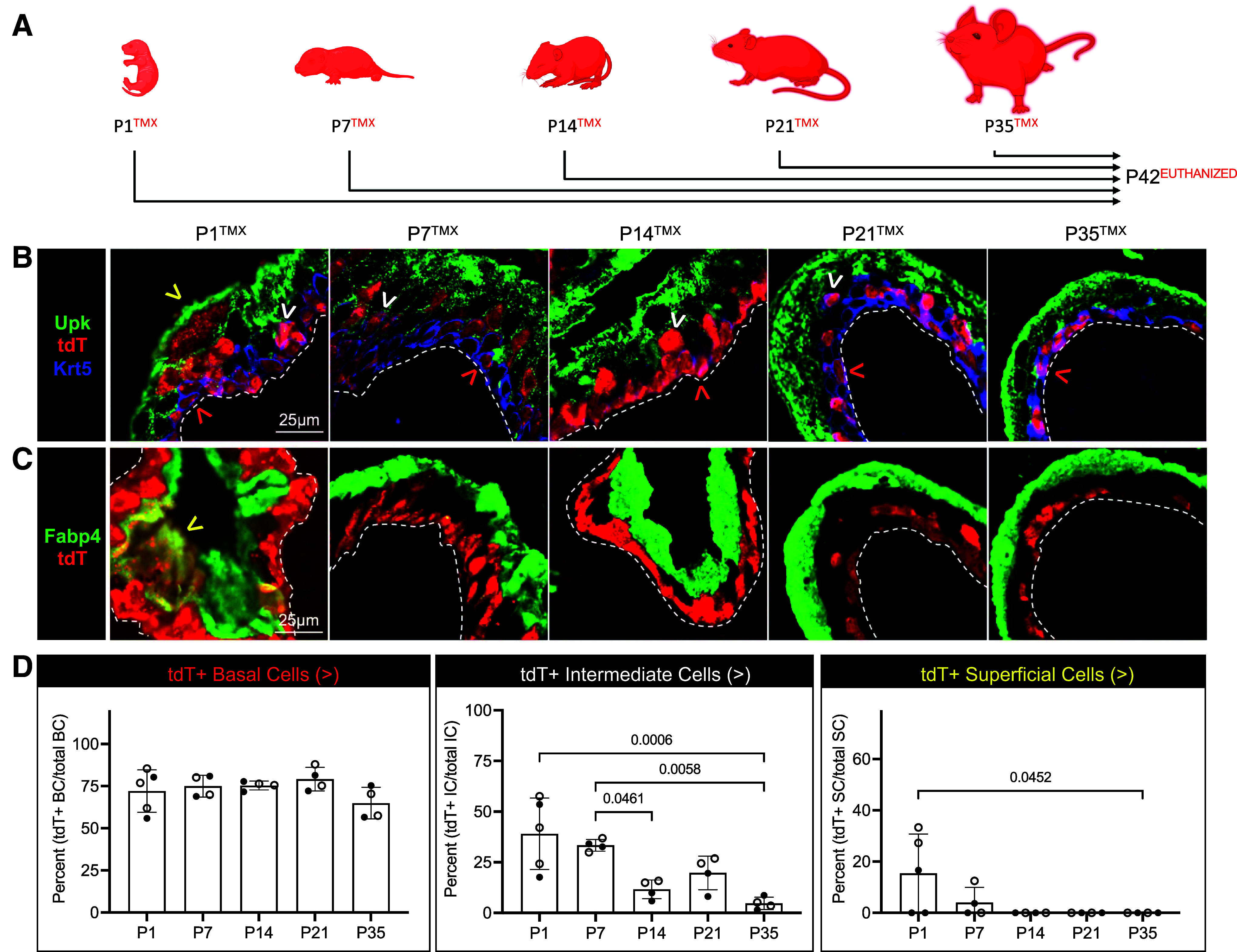
K5+ cells exhibit age-restricted intermediate and superficial cell-forming capacities. *A*: schematic representation of experimental plan showing the age tamoxifen (TMX) was administered and the age that all mice were euthanized. *B*: representative micrographs showing tdT, Krt5, and Upk expression. *C*: tdT and Fabp4 expression in urothelium in each experimental condition. White dashed line: urothelium basement membrane; red arrowhead: tdT+ basal cell; white arrowhead: tdT+ intermediate cell; yellow arrowhead: tdT+ superficial cell. *D*: graphs showing the percent tdT+ basal, intermediate, and superficial cells in each experimental condition. Bars: mean; error bars: SD; *P* values: one-way ANOVA with Šídák’s multiple comparisons test. P1→P2 (*n* = 5 mice), P7→P8 (*n* = 4 mice), P14→P15 (*n* = 4 mice), P21→P22 (*n* = 4 mice), P35→P36 (*n* = 4 mice). Closed circles: male; open circles: female. Krt5, keratin 5.

Next, we determined the fate of basal K5-UCs following their labeling at each temporal stage by evaluating the expression of tdT in adult (P42) basal cells (Krt5+, Upk−, Fabp4− within the basal layer), intermediate cells (Krt5±, Upk±, Fabp4− within intermediate layers), and superficial cells (Krt5−, Upk+, Fabp4+ in superficial layer) (Fig. 2, Supplemental Fig. S2, and Table 1). As expected, the majority (74.12% ± 6.56) of basal cells contained the indelible tdT reporter. Intermediate cells expressed tdT from each temporal stage; however, neonatal (P1, P7) K5-UCs demonstrated significantly greater intermediate cell formation than juvenile and adult (≥P14) K5-UCs. Superficial cells contained tdT from the P1 stage, but not beyond P7 (Fig. 2, Supplemental Fig. S2, and Table 1). These data demonstrate that basal K5-UCs possess age-delimited intermediate and superficial cell-forming capacities, with neonatal (P1–P7) basal K5-UC exhibiting the highest potential.

**Table 1.** Fate of K5-UCs during homeostasis

Evaluation	tdT+ B Cells	tdT+ I Cells	tdT+ S Cells
P1^TMX^ → P42^EUTHANIZED^	72.09 ± 12.60%	39.03 ± 17.61%	15.45 ± 15.32%
P7^TMX^ → P42^EUTHANIZED^	75.00 ± 6.47%	33.37 ± 2.89%	4.05 ± 5.89%
P14^TMX^ → P42^EUTHANIZED^	75.38 ± 2.632%	11.61 ± 4.64%	0%
P21^TMX^ → P42^EUTHANIZED^	79.19 ± 7.00%	19.74 ± 8.31%	0%
P35^TMX^ → P42^EUTHANIZED^	64.96 ± 9.39%	4.69 ± 3.03%	0%

Values represent means ± SD. K5-UCs, K5-expressing urothelial cells.

### The Neonatal and Juvenile K5-UC Lineage Contributes to Urothelial Restoration Following Cyclophosphamide-Induced Urothelial Injury

**Figure 3.**
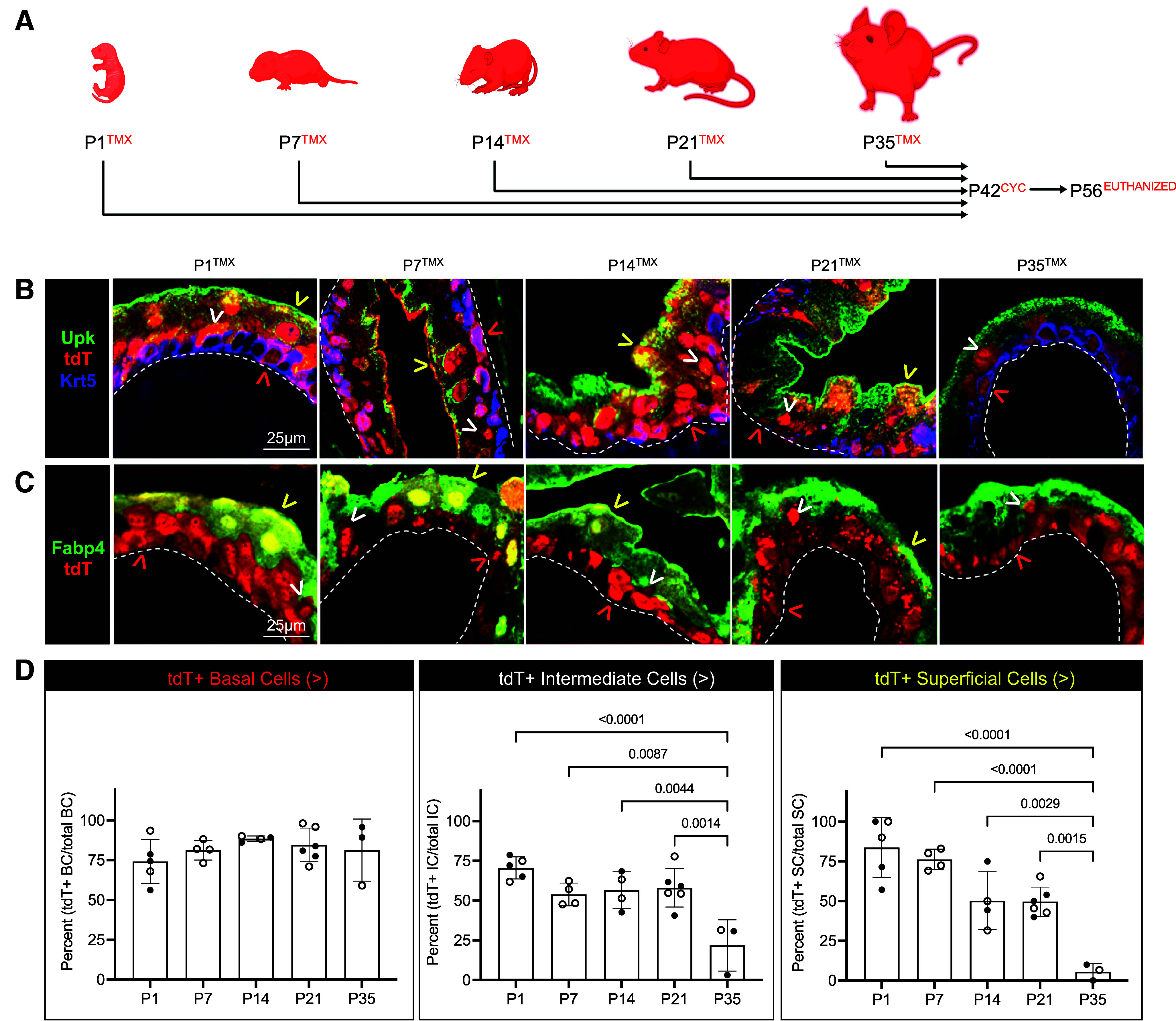
Neonatal and juvenile K5-UC lineage contributes to urothelial restoration following cyclophosphamide-induced urothelial injury. *A*: schematic representation of experimental plan showing the age tamoxifen (TMX) was administered, the age that all mice received cyclophosphamide (CYC), and the age that all mice were euthanized. *B*: representative micrographs showing tdT, Krt5, and Upk expression. *C*: tdT and Fabp4 expression in urothelium in each experimental condition. White dashed line: urothelium basement membrane; red arrowhead: tdT+ basal cell; white arrowhead: tdT+ intermediate cell; yellow arrowhead: tdT+ superficial cell. *D*: graphs showing the percent tdT+ basal, intermediate, and superficial cells at each stage. Bars: mean; error bars: SD; *P* values: one-way ANOVA with Šídák's multiple comparisons test. P1→P2 (*n* = 5 mice), P7→P8 (*n* = 4 mice), P14→P15 (*n* = 4 mice), P21→P22 (*n* = 6 mice), P35→P36 (*n* = 3 mice). Closed circles: male; open circles: female. K5-UC, K5-expressing urothelial cell; Krt5, keratin 5.

Cyclophosphamide (CYC) initiates a sequence of events consisting of loss of superficial cells 2 h after treatment, proliferation that peaks 2 days (2d) after injury, hyperplasia 3d after injury, evidence of superficial cell regeneration 7d after injury, and urothelial restoration 14–28d after injury (4, 5, 34) (Supplemental Fig. S3*A*) Although we observed increased and stratified K5-UCs at 2d, K14-UCs were strictly observed in the basal urothelial cell layer (Supplemental Fig. S3, *B* and *C*). Intermediate cells are reportedly superficial cell progenitors following a single round of 150 mg/kg CYC, while multiple rounds of CYC treatment engage K5+ basal cells for repair (4). Based on our lineage analysis results, we hypothesized that K5-UCs or their intermediate cell derivatives are the reparative progenitor. Therefore, we induced Cre;LoxP recombination at P1, P7, P14, P21, and P35 and then administered CYC or saline at P42. We evaluated the bladder urothelium 14d after CYC (Fig. 3, Supplemental Fig. S4, and Table 2). More than half of intermediate cells expressed tdT in P1, P7, P14, and P21 tamoxifen induction stages, while only 21.80% of intermediate cells expressed tdT from the P35 stage. Similarly, tdT was expressed by more than half of reconstituted superficial cells (P1–P21 stages) except for P35-labeled K5-UCs, which demonstrated a sharp reduction in tdT+ superficial cells (Fig. 3, Supplemental Fig. S4, and Table 2). These data indicate that neonatal (P1 and P7) and juvenile (P14 and P21) K5+ basal cells or their intermediate cell derivatives reconstitute the urothelium following CYC injury. Adult (P35) K5+ basal cells do not restore superficial cells following a single round of CYC-induced urothelial injury, which is consistent with other reports (4, 5).

**Table 2.** Fate of K5-UCs during tissue repair

Evaluation	tdT+ B Cells	tdT+ I Cells	tdT+ S Cells
P1^TMX^ → P42^CYC^ → P56^EUTHANIZED^	74.19 ± 13.74%	70.57 ± 6.95%	83.71 ± 18.89%
P7^TMX^ → P42^CYC^ → P56^EUTHANIZED^	81.28 ± 6.15%	53.87 ± 7.16%	76.19 ± 6.47%
P14^TMX^ → P42^CYC^ → P56^EUTHANIZED^	88.47 ± 1.72%	56.48 ± 11.62%	50.21 ± 18.24%
P21^TMX^ → P42^CYC^ → P56^EUTHANIZED^	84.58 ± 10.58%	58.08 ± 12.11%	49.62 ± 9.24%
P35^TMX^ → P42^CYC^ → P56^EUTHANIZED^	81.34 ± 19.53%	21.80 ± 16.17%	5.56 ± 5.10%

Values represent means ± SD. K5-UCs, K5-expressing urothelial cells.

### K5-UCs Exhibit Temporally Distinct Transcriptomes

**Figure 4.**
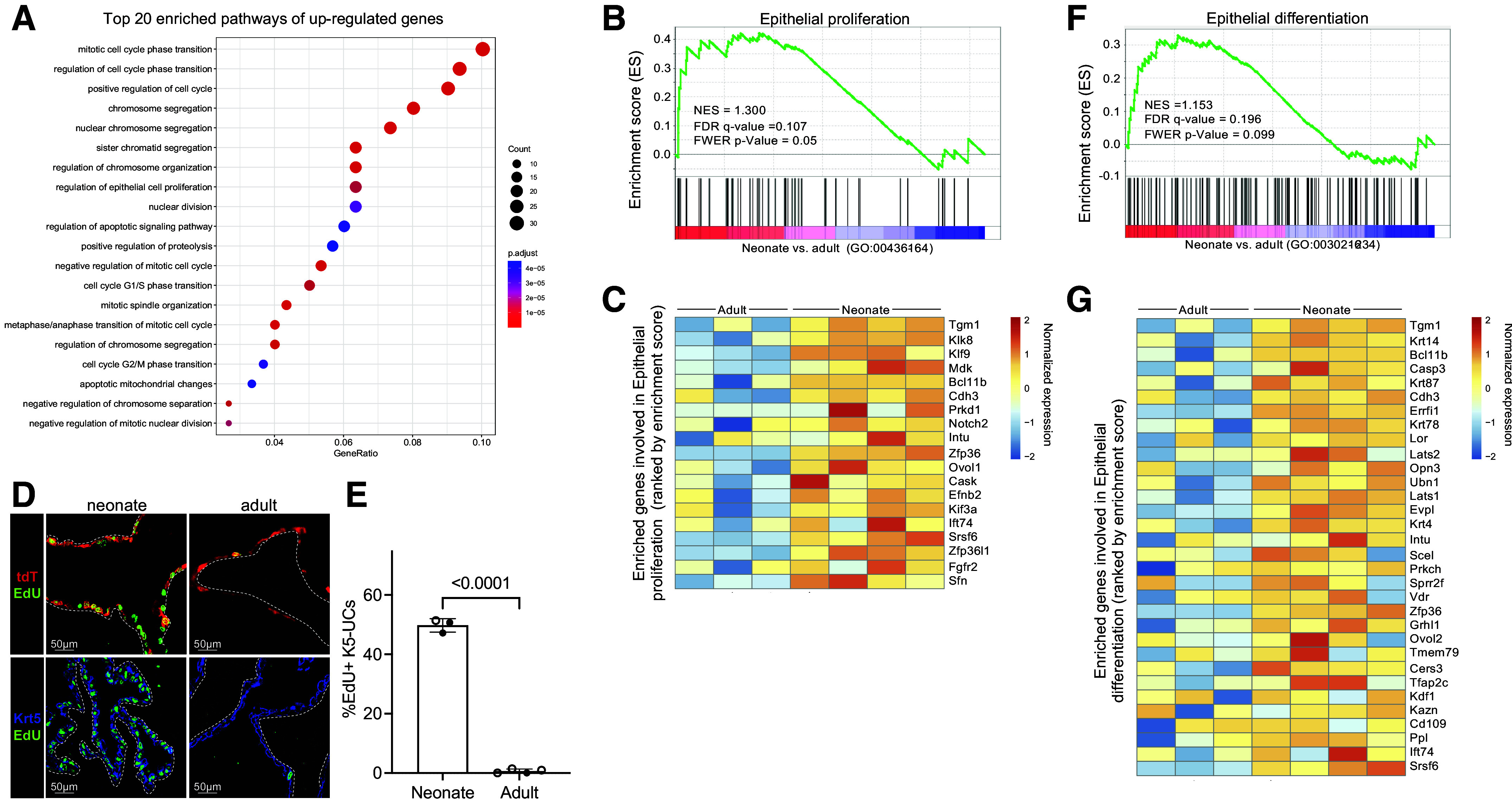
K5-UCs exhibit temporally distinct transcriptomes. *A*: top 20 enriched biological processes of upregulated genes in neonatal K5-UCs. *B*: gene set enrichment analysis for genes involved in epithelial proliferation (keratinocyte proliferation: GO:0043616) between neonates and adults. *C*: heatmap of differentially expressed genes involved in epithelial proliferation ranked by enrichment scores. *D*: representative micrographs showing tdT and EdU localization (*top*) and Krt5 and EdU localization (*bottom*) in neonate (P7) and adult (P35) bladders. White dashed line: urothelium basement membrane. *E*: graph showing the proliferative index in neonate (P7) and adult (P35) K5-UCs. Bars: mean; error bars: SD; *P* values: unpaired two-tailed *t* test. Closed circles: male; open circles: female. *F*: gene set enrichment analysis for genes involved in epithelial differentiation (keratinocyte differentiation: GO:0030216) between neonates and adults. *G*: heatmap of differentially expressed genes involved in epithelial differentiation ranked by enrichment scores. EdU, 5-ethynyl-20-deoxyuridine; K5-UCs, K5-expressing urothelial cells; Krt5, keratin 5.

Given the distinct progenitor capacities observed by neonatal and adult K5-UCs, we sorted K5-UCs from P7 and P35 bladders and used RNAseq to determine whether functional differences could be explained by gene expression. Principal component analysis identified distinct clusters between neonate and adult samples, and substantiated removal of one adult outlier (Supplemental Fig. S5*A*). Our RNA-seq analysis unveiled 645 differentially expressed genes (DEGs) in neonates, with 311 upregulated and 334 downregulated (Supplemental Fig. S5, *B* and *C*). Gene Ontology (GO) analysis demonstrated that the upregulated genes in neonates were predominantly associated with cellular proliferation (17 of top 20 GO terms), whereas downregulated genes were predominantly associated with metabolic processes (Fig. 4*A* and Supplemental Fig. S5*D*). Gene set enrichment analysis highlighted the upregulation of genes associated with epithelial proliferation (Fig. 4, *B* and *C*). An in-depth examination of UC markers found that neonatal K5-UCs exhibited heightened expression levels of certain basal (*Krt15* and *Krt14*) and cycling (*Top2a*, *Mki67*, and *Birc5*) genes (Supplemental Fig. S5*E*) (35, 36). Transcription factor analyses revealed higher expression levels of protooncogenes in neonatal K5-UC, including Fos, Jun, Myc, and Egr1, indicating their potential roles in promoting cell proliferation (Supplemental Fig. S5*F*). EdU incorporation assays confirmed increased K5-UC proliferation in neonates compared with adults (Fig. 4, *D* and *E*). Gene set enrichment analysis also highlighted the upregulation of genes associated with epithelial differentiation (Fig. 4, *F* and *G*) in neonatal K5-UCs, where higher levels of intermediate and superficial cell markers (*Upk2a*, *Upk1a*, *Krt18*, *Krt7*, and *Krt19*) indicated an increased capacity for cell differentiation (Supplemental Fig. S5*E*) (35). This analysis illuminates the molecular distinctions that underlie the enhanced progenitor potential of neonatal K5-UCs cells compared with their adult counterparts, shedding light on their unique biological properties.

### K5-UC Organoid-Forming Capacities Are Temporally Governed

Following our observation that the K5+ basal cell lineage gives rise to intermediate and superficial cells in a temporally restricted manner in vivo, and that pathways associated with cellular proliferation are enriched in neonatal K5-UCs, we used organoid assays to determine whether neonate progenitor capacity is maintained in vitro.

We confirmed that single tdT^K5^-UCs formed organoids in a clonal manner (tdT observed throughout the multicellular organoids, Supplemental Fig. S6*A*). Furthermore, when labeled with urothelial markers, organoids were found to organize into multicellular structures, with Krt5 expression around the perimeter (basal) and Upk expression around the lumen (apical), indicating that the tdT^K5^-UCs also self-organized and differentiated (Supplemental Fig. S6*B*).

**Figure 5.**
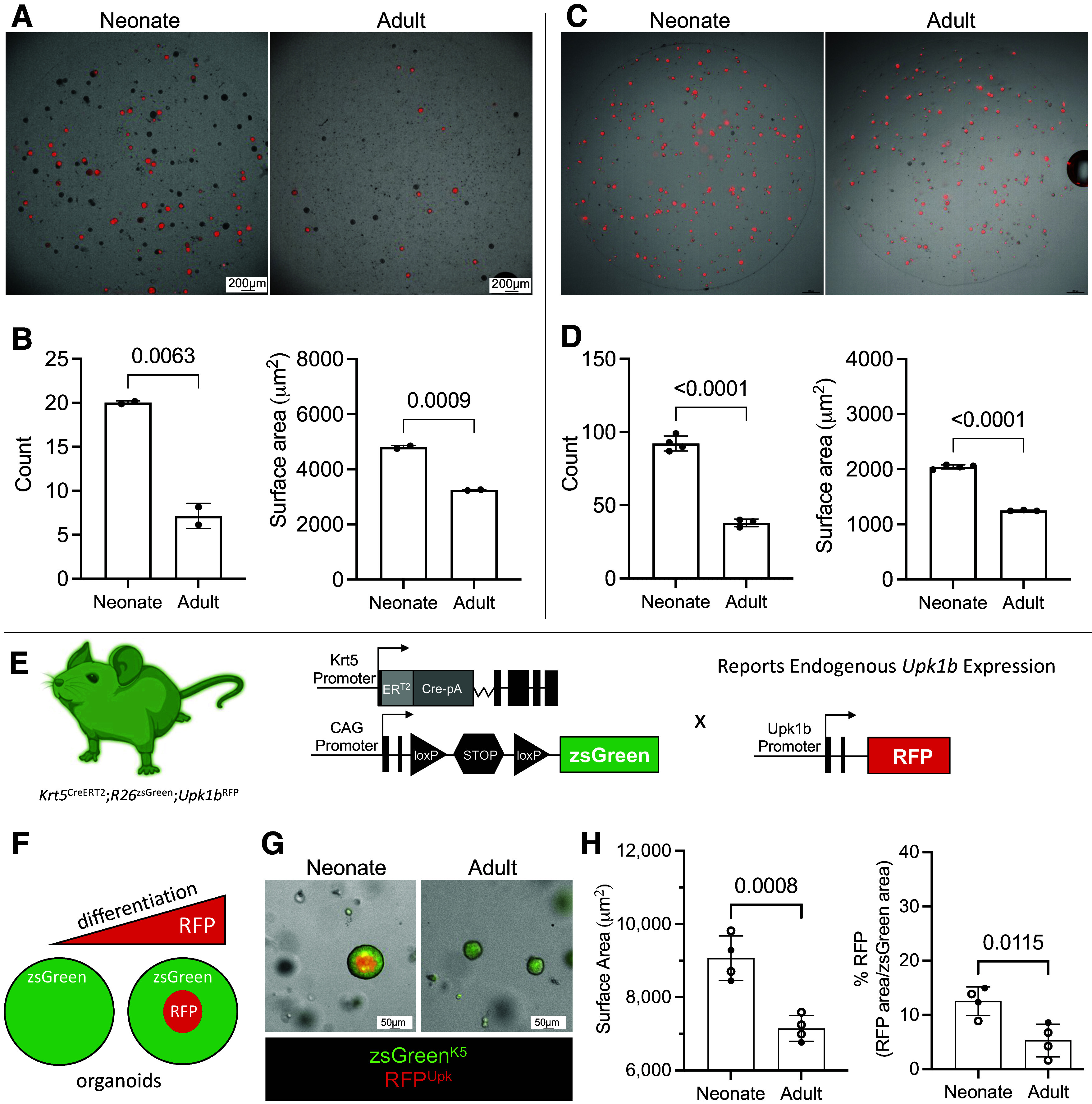
Neonatal K5-UCs maintain increased progenitor capacity in vitro. *A*: representative micrographs showing neonate (P7) and adult (P35) urothelium-derived organoids at 5 days. *B*: graphs showing the number (count) and size (surface area) of tdT+ organoids at 5 days. Bars: mean; error bars: SD; *P* values: unpaired two-tailed *t* test. Neonate (*n* = 2 Matrigel domes, sexes pooled), adult (*n* = 2 Matrigel domes, sexes pooled). *C*: representative micrographs showing neonate (P7) and adult (P35) urothelium-derived organoids established from FACS-enriched tdT+ urothelial cells at 5 days. *D*: graphs showing the number (count) and size (surface area) of the organoids established from FACS-enriched tdT+ urothelial cells at 5 days. Bars: mean; error bars: SD; *P* values: unpaired two-tailed *t* test. Neonate (*n* = 5 Matrigel domes, sexes pooled), adult (*n* = 3 Matrigel domes, sexes pooled). *E*: schematic of the *Krt5*^CreERT2^;*R26*^zsGreen^;*Upk1b*^RFP^ mouse and resultant dual reporter organoid model. *F*: schematic showing that organoids developed from *Krt5*^CreERT2^;*R26*^zsGreen^;*Upk1b*^RFP^ mice are zsGreen+ (K5-UC lineage) and those that differentiate express RFP from the endogenous *Upk1b* promoter in their interior (apical). *G*: representative micrographs showing neonate (P7) and adult (P35) urothelium-derived organoids at 7 days. zsGreen: Krt5 lineage, RFP: *Upk1b* expression. *H*: graphs showing the size (surface area) and percent RFP expressed by zsGreen+ organoids (RFP area/zsGreen area). Bars: mean; error bars: SD; *P* value: unpaired two-tailed *t* test. Neonate (*n* = 4 mice), adult (*n* = 4 mice). Closed circles: male; open circles: female. K5-UCs, K5-expressing urothelial cells; Krt5, keratin 5.

Next, we compared organoid-forming capacity of neonate and adult K5-UCs. We found that tdT+ urothelial cells from neonatal (P7) mice formed more organoids and larger organoids than adult (P35) mice (Fig. 5, *A* and *B*, and Supplemental Fig. S6, *C* and *D*). To ensure our results were due to age-related differences in K5-UCs rather than different densities of K5-UCs at each age, we enriched K5-UCs by FACS and plated an equivalent number of tdT+ cells from neonate and adult bladders. Although the size of the organoids remained larger when comparing neonate and adult organoids, FACS enrichment of tdTs led to the formation of nearly fivefold more organoids compared with urothelium that was directly plated (Fig. 5, *C* and *D*).

Next, we generated a dual reporter mouse (*Krt5*^CreERT2^;*R26*^zsGreen^;*Upk1b*^RFP^) to visualize K5-UC differentiation in organoid assays (Fig. 5*E*). These mice permanently express zsGreen in K5-UCs and their derivatives in a TMX-inducible manner, while RFP expression is regulated by the endogenous *Upk1b* promoter (Fig. 5, *E* and *F*). We again confirmed that organoids formed from neonatal K5-UCs were larger than adults (Fig. 5, *G* and *H*), and observed increased RFP in organoids formed from neonatal K5-UCs compared with adults (Fig. 5, *G* and *H*). These data confirm our in vivo observations and RNAseq results and underscore the heightened progenitor capacity in neonate K5-UCs.

### FGF7-Induced K5-UC Proliferation Restores Progenitor Potential In Vivo

**Figure 6.**
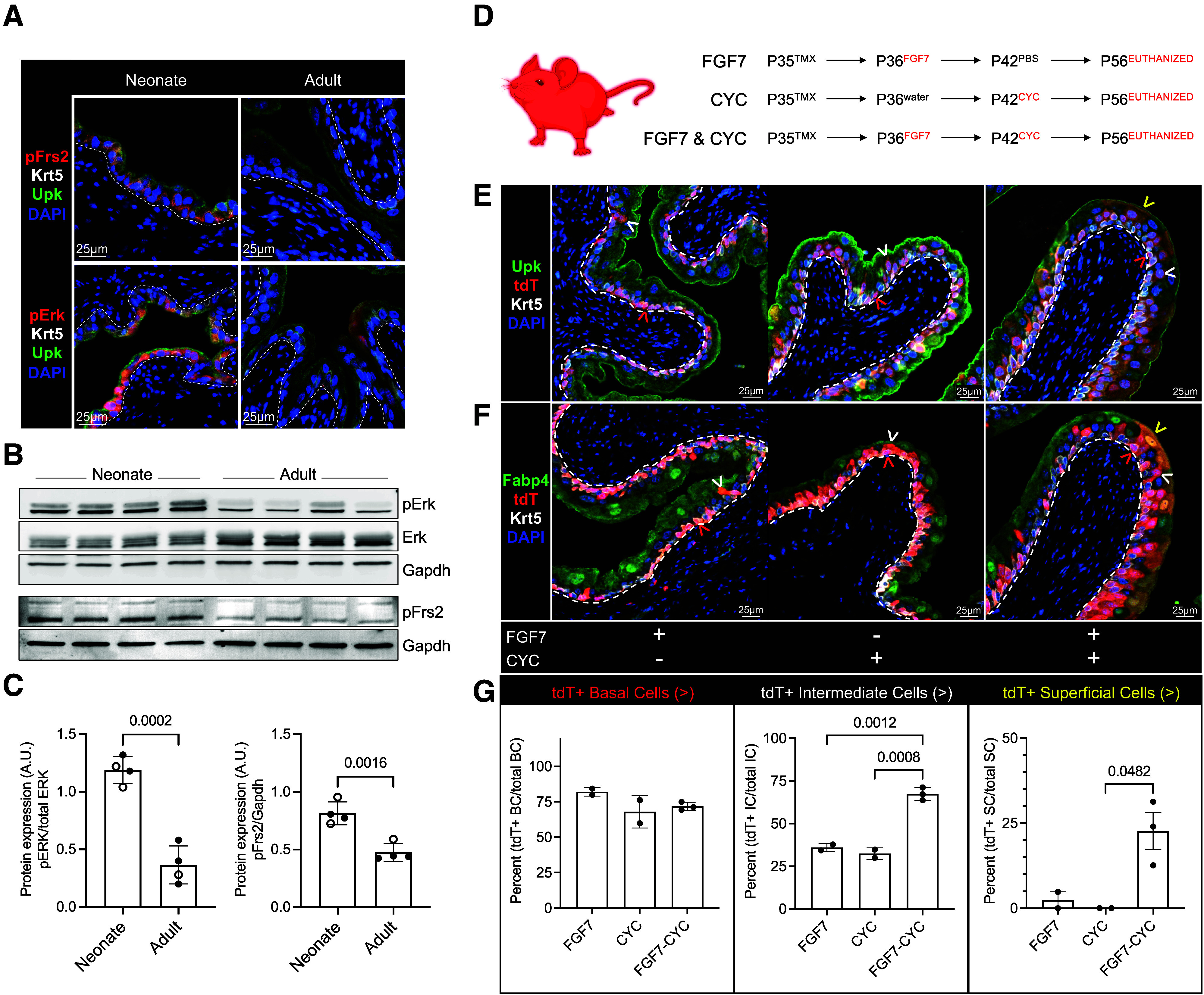
FGF7 restores the progenitor function of adult K5-UCs during CYC injury. *A*: representative micrographs showing pFrs2, Krt5, Upk, and DAPI (*top*) and pErk, Krt5, Upk, and DAPI (*bottom*) expression in neonate (P7) and adult (P35) bladder urothelium. White dashed line: urothelium basement membrane. *B*: immunoblot showing pErk1/2 (pErk), total Erk1/2 (Erk), pFrs2, and Gapdh expression in neonate (P7) and adult (P35) bladder urothelium. *C*: graph showing pErk/total Erk and pFrs2/Gapdh levels as measured by densitometry. Error bars: SD; *P* values: unpaired two-tailed *t* test. Neonate (*n* = 4 mice), adult (*n* = 4 mice). Closed circles: male; open circles: female. *D*: schematic representation of experimental plan showing the age tamoxifen (TMX), rhFGF7 (FGF7), cyclophosphamide (CYC), or their carrier controls (water or PBS) were administered and the age that mice were euthanized. *E*: representative micrographs showing Upk, tdT, Krt5, and DAPI expression in each experimental condition. *F*: representative micrographs showing Fabp4, tdT, Krt5, and DAPI expression in each experimental condition. White dashed line: urothelium basement membrane. Red arrowhead: tdT+ basal cell; white arrowhead: tdT+ intermediate cell; yellow arrowhead: tdT+ superficial cell. *G*: graphs showing the percent tdT+ basal, intermediate, and superficial cells in each condition. Bars: mean; error bars: SD; *P* values: one-way ANOVA with Tukey’s multiple comparisons test. CYC (*n* = 2 mice), FGF7 (*n* = 2 mice), FGF7-CYC (*n* = 3 mice). Closed circles: male; open circles: female. K5-UCs, K5-expressing urothelial cells; Krt5, keratin 5.

K5-UC proliferation and progenitor capacity are inversely correlated with age, leading us to hypothesize that induced proliferation may restore progenitor potential of adult K5-UCs. Transcriptional profiling identified increased *Fgfr2* in neonate K5-UCs (Fig. 4*C*), the urothelial receptor for the potent mitogen Fgf7. We found increased pFrs2 and pErk1/2 expression in neonatal K5-UCs, suggesting activation of the proliferative Fgf7-Fgfr2 signaling pathway (Fig. 6, *A*–*C*) (37). We thus investigated whether recombinant FGF7 could restore lineage-restricted adult K5-UCs (34, 38, 39).

First, we determined the impact of FGF7 on adult K5-UC proliferation. Twenty-four hours after FGF7 administration, we found a statistically significant increase in urothelial cell proliferation compared with water-treated controls (FGF7: 30.10% ± 1.44, water: 1.46% ± 0.79) (Supplemental Fig. S7, *A* and *B*). We observed that 37.68% ± 1.27 of K5-UCs had incorporated EdU, and that K5-UCs were the dominant proliferative urothelial cell (93.36% ± 4.72) following FGF7 treatment (Supplemental Fig. S7*B*). These data confirm that FGF7 is an effective and highly specific tool to proliferate K5-UCs and evaluate the impact of proliferation on the K5-UC lineage.

Our data indicate that P35 K5-UCs rarely form intermediate or superficial cells, and that P35 labeled K5-UCs rarely contribute to superficial cell restoration following CYC-induced urothelial injury (Figs. 2 and 3). Given that adult urothelium has a low mitotic index, we administered FGF7 to *Krt5*^CreERT2^;*R26*^tdT^ mice following P35 TMX administration and determined the impact of FGF7 on the K5-lineage with and without CYC-induced urothelial injury (Fig. 6*D*). FGF7 did not lead to a significant increase in intermediate or superficial cell formation in the absence of urothelial injury; however, we observed statistically significant increases in intermediate and superficial cell formation when FGF7 was administered prior to CYC-induced urothelial injury (Fig. 6, *E*–*G*). Thus, FGF7-induced K5-UC proliferation restored the adult K5-UC reparative capacity.

## DISCUSSION

The progenitor responsible for bladder urothelium repair has been the focus of many investigations. By varying the type (chemical vs. bacterial vs. surgical) and chronicity (acute vs. repeated) of injury, investigators have uncovered context-dependent urothelial repair populations [reviewed in Jackson et al. (14)]. Accounting for the dynamic formation of bladder urothelium during postnatal life and its highly stable and quiescent existence in adulthood, we set out to define the fate of K5-UCs labeled at various developmental stages to define how the Krt5 lineage contributes to postnatal maturation and repair. Although adult basal K5-UCs cells rarely give rise to intermediate cells, we report that neonatal and juvenile Krt5+ basal cells give rise to adult intermediate cells. Our data show that adult intermediate cells derived from the neonatal and juvenile Krt5-lineage reconstitute superficial cells damaged by cyclophosphamide-induced injury. By accounting for age, our findings unite what is considered a “basal cell versus intermediate cell progenitor theory,” and support a linear model of urothelial formation and regeneration (basal → intermediate → superficial cell).

Label retaining cell (LRC) assays and inducible Cre drivers have been widely applied to investigate the urothelial progenitor [reviewed in Jackson et al. (14)]. These studies support basal or intermediate cell progenitor cells. We and others note that the Krt5 antigen is detected in basal cells and a subset of intermediate cells in the bladder urothelium (5). However, we demonstrated here that a single ip dose of TMX to *Krt5*^CreERT2^ mice induces recombination (via tdT expression) predominantly in basal Krt5+ cells (Fig. 1). Although other studies used multiple doses of TMX in adult mice, we specifically designed our experiments so that we could map the fate of basal Krt5+ cells. Indeed, we achieved high-specificity tdT labeling predominantly in basal K5-UCs, which allowed us to interpret a temporally regulated fate for the basal fraction of K5-UCs.

Adult bladder urothelium is nearly quiescent, and urothelial cells exhibit a 3–6 mo or greater turnover rate (2, 6). However, the embryonic and postnatal bladder is highly proliferative (7, 20). Interestingly, the urothelium experiences desquamation and apoptosis after birth, which requires a postnatal urothelial restoration (19). We confirmed neonatal bladders were highly proliferative compared with nearly quiescent adult bladders in this study, and that K5-UCs were the most dominant proliferative cell in neonates. Colopy et al. showed that P1 bladder urothelium exhibits the greatest proliferative capacity (30.4%), with >95% of proliferating cells in the basal layer (20). LRC assays demonstrated that when BrdU was pulsed at P1, LRCs were dispersed in basal, intermediate, and superficial layers after a 1-mo chase, evidencing these cells were progeny of a basal cell progenitor. Indeed, we found that the P1 tdT-labeled K5-UCs exhibit the greatest progenitor capacity, with tdT identified in adult basal, intermediate, and rare superficial cells. When BrdU was administered at P7, LRCs were identified in basal and intermediate layers 1 mo later (20). Likewise, we found that P7 K5-UCs gave rise to adult basal and intermediate cells. Lastly, when BrdU was administered at P14, LRCs were predominantly in the basal layer 1 mo later (20). Our data recapitulate this finding as well, with a significant reduction in the percentage of adult intermediate cells derived from P14-labeled K5-UCs. By showing the contributions to intermediate cells (and rare superficial cells) arising from the neonatal Krt5 basal cells, our findings confirm postnatal restoration dynamics and highlight the stable nature of mature urothelium, which exhibits little progenitor activity.

Chemical, bacterial, and surgical insults have been used to investigate progenitor activity during urothelial repair. Evidence supporting contributions of basal or intermediate cells during urothelial restoration is seemingly context-dependent. Shin et al. reported that Shh+ cells (basal + intermediate cell) reconstitute the bladder following chronic bacterial injury (12). Gandhi et al. reported that Upk3a+ cells (intermediate cell) reconstituted CYC-injured urothelium (5). Papafotiou et al. found that Krt14+ (basal cell) and K5 (basal cell) restored all urothelial layers following a single dose of CYC (13). Schafer et al. reported that Upk2+ cells (intermediate cell) reconstituted bladder urothelium following focal mucosal injury, while Krt5 (basal cell) reconstituted urothelium following augmentation cystoplasty (40). Wang et al. reported that Upk3a+ (intermediate cell) reconstitute urothelium following exposure to a single round of CYC, while Krt5 (basal cell) contribute to urothelial restoration following five doses of CYC-induced urothelial injury (4). Technical and methodological differences may explain divergent observations between our findings and those of other K5-lineage analysis studies. First, we used CYC at 150 mg/kg as was the case for Wang et al. and Gandhi et al., while Papafotiou et al. administered 250 mg/kg CYC (4, 5, 13). Papafotiou found a far greater contribution of K5-UCs to I (51%) and S (27%) cells with the higher concentration CYC, while Wang et al. and our study showed a lower contribution to I (20 + 16 = 36%) and S (7.5%) cells, which more closely matched ours (P35 K5-UC derivatives: 22% I, 6% S). However, our lineage labeling strategy was strict, using only 1 dose of TMX in an effort to strictly label K5 basal cells. Furthermore, differences in the age at which TMX was introduced could be a factor. Our study showed that adult I cells were formed by P1–P21 K5-UCs at higher rates than P35 K5-UCs, and that those P1–P21-labeled K5-UCs contributed significantly to S cell formation following CYC. Given that adult urothelium is highly stable during homeostasis, it is reasonable to expect that adult (>P35) K5-UCs rarely form progeny following an acute injury. Instead, our data lead us to conclude that neonate or juvenile (P1-P21) derived I cells contribute to urothelial restoration following acute injury.

Whether basal K5-UCs (or a subset of Krt14+ K5-UCs) represent the bladder stem cell, adult intermediate cells certainly contribute to restoring the urothelium during injury, and the concept of stem/progenitor may be regarded as simply semantics. Although the exact contribution of intermediate cells to homeostasis or repair is unclear, future studies that deploy inducible *Sprr2f* or *Ugt2b34* Cre drivers may shed light on intermediate cell lineage dynamics (35). The similarities of a slow turnover bladder urothelium to a high turnover epithelium such as skin or gut are unclear, yet perhaps the intermediate urothelial cell can be likened to transit-amplifying cells (TACs) described in other tissues. TACs are defined as stem cell-derived, nondifferentiated proliferative multipotent cells capable even of regulating stem cell proliferation (41). Given our observation that bladder intermediate cells are derived from P1 to P21 K5-UCs, our observations support a linear model of urothelial formation and regeneration (basal → intermediate → superficial cell), where basal cells serve as the stem cell and basal cell-derived intermediate cells serve as injury responsive transit-amplifying cells. Papafotiou et al. seemingly suggested a similar theory where they proposed that if a stem cell dedicated to superficial cell regeneration exists, “they can only be short-term urothelial stem cells (USCs) sufficient to regenerate mildly injured bladders” (13). As was shown by Wang et al., only after multiple rounds of injury (called chronic) does the adult K5-UC contribute to urothelial restoration, likely after intermediate cells (which we call the putative TACs) are depleted (4). Indeed, we believe our findings unite previously conflicting observations and reveal context-dependent observations that require a more complex regulation of TAC versus stem cell activation for restoration. Nonetheless, the full repertoire of temporally permissive K5-UCs progeny is important to understand when investigating urothelial generation, regeneration, and eventual tissue engineering studies.

Organoid assays are robust model system to investigate urothelial progenitor characteristics (12, 13, 42, 43). These assays permit investigation of cells isolated from different contexts (ages) to be cultured in identical microenvironments (Matrigel + media). We used organoid models not only to validate our in vivo fate mapping observations, but also to determine whether superior progenitor function was intrinsic to neonatal K5-UCs. Papafotiou et al. observed tdT+ Krt14+ basal cells formed more organoids than tdT(−) (13), and Santos et al. found that cd49f^high^ basal cells had superior organoid-forming capacity compared with low cd49f^low^ urothelial cells (43). Our observation that tdT+ K5-UCs formed more organoids than tdT(−) cells confirm those reports. Elegant work by Mullenders et al. described methods to establish primary murine basal cell organoids and suprabasal bladder organoids (42). Although different enzymatic and mechanical enrichment strategies established organoids with divergent “basal” and “luminal” profiles, the absence of a reporter makes it difficult to discern the precise contributions of each lineage. Ultimately our goal in using organoid models was to validate whether neonatal K5-UCs outperformed adult K5-UCs as progenitors. When tdT+ K5-UCs from neonate and adult mice were placed in identical in vitro conditions, neonatal K5-UCs formed more organoids than their adult counterparts, which indicate that a greater proportion of neonatal K5-UCs have progenitor function. Furthermore, we observed that neonate K5-UCs formed larger organoids, which indicates that they have a greater self-renewal or proliferative capacity than adults K5-UCs, which is supported by our RNAseq analysis which identified increased proto-oncogene activity and upregulation of proliferation-associated genes in neonates. Lastly, our dual reporter organoid model established that neonate K5-UCs formed organoids that expressed more *Upk* than adult K5-UCs, which is supported by our lcRNAseq analysis which identified that several regulators of cell fate specification were activated in neonates. Future studies will use this sophisticated model to determine unique biological properties of neonate and adult K5-UCs and interrogate the precise signaling programs that govern progenitor self-renewal and differentiation.

K5-UC progenitor capacity and proliferation are inversely correlated with age. This led us to question whether application of an exogenous proliferative trigger could reengage adult K5-UC progenitor function. Fgf7 is a known urothelial mitogen (38, 39, 44), and our data demonstrate that it acts predominantly on K5-UCs and restores the capacity of adult K5-UCs to reconstitute injured urothelium. In addition to its role as a urothelial mitogen, a mechanism by which FGF7 orchestrates cytoprotection was recently discovered. Narla et al. observed that FGF7 led to activation of AKT and intermediate and superficial cell cytoprotection during CYC treatment (34). We believe that our use of FGF7 as a urothelial mitogen in this study is distinct from its cytoprotective use. Specifically, we separated FGF7 treatment and CYC administration by 1 wk, whereas its cytoprotective role was observed when FGF7 preceded CYC by just 24 h. Although we used FGF7 as a tool to proliferate K5-UCs, the proliferative impact is likely due to Erk activation (34). The precise mechanism by which FGF7 induces proliferation and releases lineage restriction in K5-UCs presents an intriguing direction for this work, which may include an investigation into whether FGF7-stimulated K5-UCs express genes reminiscent of neonate K5-UCs.

### Conclusions/Summary

By mapping the fate of postnatal K5-UCs, we revealed important linear relationships whereby K5-UCs give rise to intermediate and superficial cells at an age-restricted manner and contribute to urothelial restoration following injury. Since neonatal K5-UCs reprise their role as superior progenitors in vitro and display distinct transcriptional signatures, this suggests that progenitor function is at least partially cell-intrinsic. However, the urothelium progenitor niche cannot be overlooked, since FGF7 can rescue the progenitor function of adult K5-UCs. Additional studies should investigate the full repertoire of cell-cell, mesenchyme-derived, or urine-derived factors that may vary with age. The novel dual reporter organoid system developed here will be important to the identification of additional progenitor-influencing signals.

## DATA AVAILABILITY

Data will be made available upon reasonable request.

## SUPPLEMENTAL DATA

10.6084/m9.figshare.24500548 Supplemental Figs. S1–S7: https://doi.org/10.6084/m9.figshare.24500548; Supplemental Tables S1 and S2: https://doi.org/10.6084/m9.figshare.24500545.

## GRANTS

This work was supported by National Institute of Diabetes and Digestive and Kidney Diseases Grants F32DK115085 and K01DK126991 (to A.R.J.) and R01DK125469 (to B.B.) as well as intramural support from the Abigail Wexner Research Institute (to A.R.J.). Research reported in this publication was supported by The Ohio State University Comprehensive Cancer Center and the National Institutes of Health under Grant P30CA016058. We thank Genomics at The Ohio State University Comprehensive Cancer Center (Columbus, OH) for lcRNAseq support. Palifermin (Kepivance) was generously provided by Swedish Orphan Biovitrum AB (SOBI, Solna, Sweden).

## DISCLOSURES

No conflicts of interest, financial or otherwise, are declared by the authors.

## AUTHOR CONTRIBUTIONS

B.B. and A.R.J. conceived and designed research; M.E.-H., F.R.-T., K.M.G., B.L., M.K., and A.R.J. performed experiments; M.E.-H., F.R.-T., X.W., and A.R.J. analyzed data; B.B., M.E.-H., F.R.-T., and A.R.J. interpreted results of experiments; A.R.J. prepared figures; A.R.J. drafted manuscript; B.B. edited and revised manuscript; B.B., M.E.-H., F.R.-T., K.M.G., B.L., M.K., X.W., and A.R.J. approved final version of manuscript.
